# Lp(a): a New Pathway to Target?

**DOI:** 10.1007/s11883-022-01060-4

**Published:** 2022-09-06

**Authors:** Nick S. Nurmohamed, Jordan M. Kraaijenhof, Erik S. G. Stroes

**Affiliations:** 1 Department of Vascular Medicine, Amsterdam UMC, University of Amsterdam, Meibergdreef 9, Amsterdam, 1105 AZ The Netherlands; 2 Department of Cardiology, Amsterdam UMC, Vrije Universiteit Amsterdam, Amsterdam, The Netherlands

**Keywords:** Lipoprotein(a), AVS, ASCVD

## Abstract

**Purpose of Review:**

Over the past decades, genetic and observational evidence has positioned lipoprotein(a) as novel important and independent risk factor for cardiovascular disease (ASCVD) and aortic valve stenosis.

**Recent Findings:**

As Lp(a) levels are determined genetically, lifestyle interventions have no effect on Lp(a)-mediated ASCVD risk. While traditional low-density lipoprotein cholesterol (LDL-C) can now be effectively lowered in the vast majority of patients, current lipid lowering therapies have no clinically relevant Lp(a) lowering effect.

**Summary:**

There are multiple Lp(a)-directed therapies in clinical development targeting *LPA* mRNA that have shown to lower Lp(a) plasma levels for up to 90%: pelacarsen, olpasiran, and SLN360. Pelacarsen is currently investigated in a phase 3 cardiovascular outcome trial expected to finish in 2024, while olpasiran is about to proceed to phase 3 and SLN360’s phase 1 outcomes were recently published. If proven efficacious, Lp(a) will soon become the next pathway to target in ASCVD risk management.

## Introduction

**Fig. 1:**
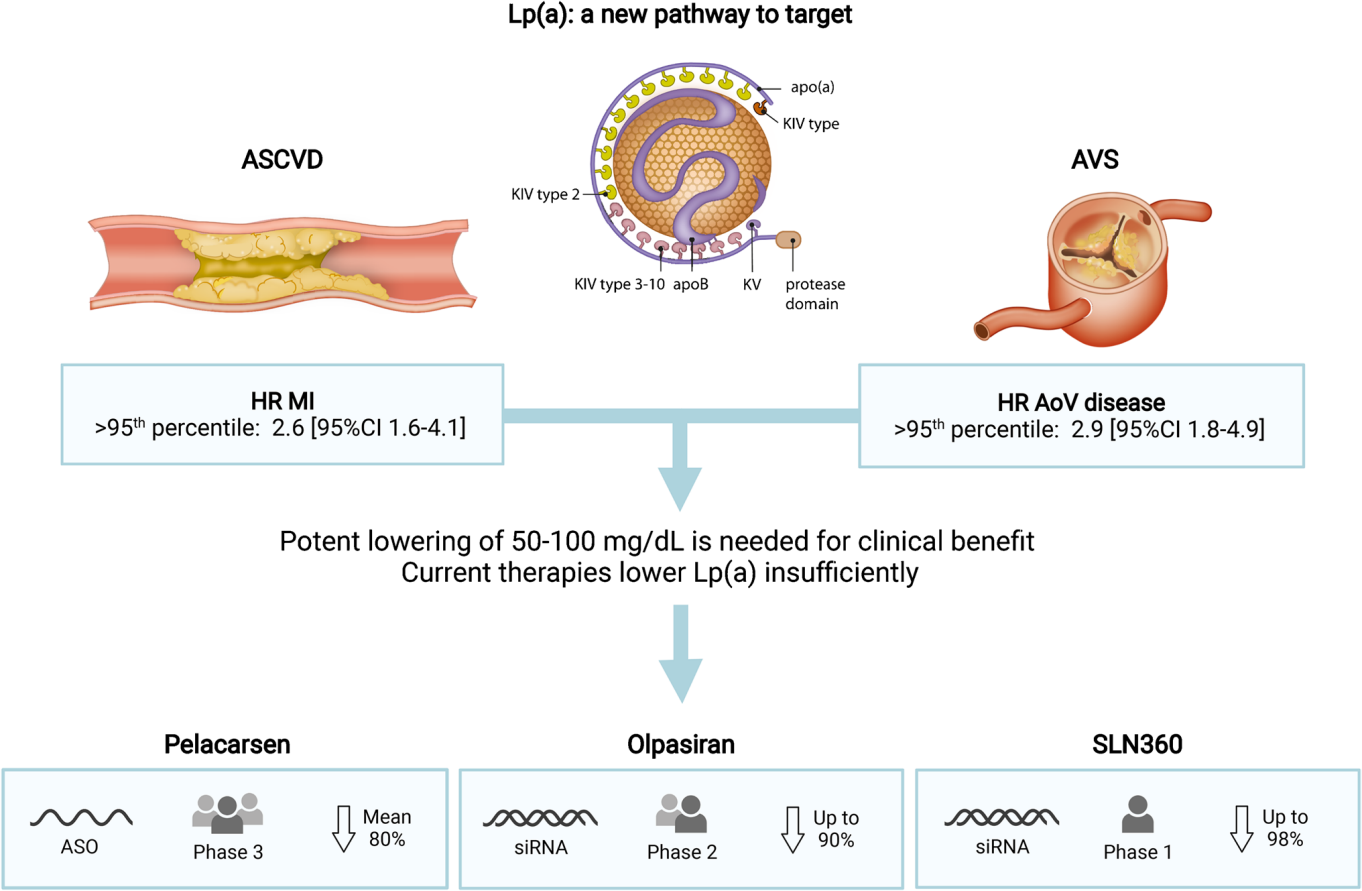
Lp(a): a new pathway to target. Overview figure illustrating the hazard ratios (HR) for myocardial infarction (MI) and aortic valve (AoV) disease associated with lipoprotein(a) (Lp[a]) levels above the 95th percentile as well as Lp(a)-directed therapeutics in clinical development. ASCVD, atherosclerotic cardiovascular disease; AVS, aortic valve stenosis; ASO, antisense oligonucleotide; siRNA, small interfering RNA. Created with BioRender.com

Lipoprotein(a) (Lp(a)) consists of an apolipoprotein B100 (apoB) particle containing cholesterol and triglycerides and is covalently bound to an apolipoprotein(a) unit. Already in the early 1970s, the first associations between Lp(a) and coronary heart disease were reported [1]. In the decades following, the lack of a reliable assay and uncertainties in Lp(a) biology relegated Lp(a) into the background of atherosclerotic cardiovascular disease (ASCVD) research. However, in the last decade, following large epidemiological, genome-wide association (GWAS) and Mendelian randomization studies combined with contemporaneous development of more reliable immunoassays, Lp(a) has been reinvented as an important ASCVD risk factor. Given the frequency of high Lp(a) levels and the previous lack of effective Lp(a) lowering therapies, the potentially modifiable Lp(a) burden could become the most important risk factor to target in the coming decade. This review addresses the emerging role of Lp(a) as potentially modifiable risk factor for ASCVD and aortic valve stenosis (Fig. 1).

## Lp(a): an Important Risk Factor for ASCVD and Aortic Valve Stenosis

Lp(a) levels are primarily (> 90%) genetically determined and are not influenced by lifestyle. Therefore, Lp(a) levels remain stable over life, in contrast to other cholesterol-carrying apoB particles such as low-density lipoprotein (LDL) particles. The Lp(a) plasma concentration is determined by two alleles of the *LPA* gene, coding for the apolipoprotein(a) molecule of the Lp(a) particle. This apolipoprotein(a) molecule is largely homologous to plasminogen and comprises a protease domain coupled to 10 types of kringle IV structures and one kringle V structure. The genetically determined size is highly variable between individuals and depends on the number of kringle IV type 2 coding repeats in the *LPA* gene. Since every individual has two copies of this gene, two different isoforms of Lp(a) are present in the plasma. In general, the Lp(a) plasma concentration is inversely correlated with the number of kringle IV repeats; hence, patients with larger Lp(a) molecules have lower plasma levels. As expected, family studies have shown a large correlation of Lp(a) levels in families. Depending on the cut-off used, up to 20% of individuals worldwide have elevated Lp(a) plasma levels. Absolute cut-off values should be cautiously interpreted, since plasma levels of Lp(a) are highly dependent upon ethnicity [2]. In a recent analysis by Mehta et al. in the MESA (Multi-Ethnic Study of Atherosclerosis), black participants had a median Lp(a) level of 35.2 mg/dl, much higher than white, Hispanic, or Chinese participants (median 13.2 mg/dl) [3]. Despite the racial differences, the ASCVD risk resulting from Lp(a) seems largely similar across different ethnicities [2].

Observational, GWAS, and Mendelian randomization evidence have clearly demonstrated that there is a log-linear relationship between plasma Lp(a) levels and ASCVD risk [4•, 5, 6]. In one of the Mendelian randomization analyses by the group of Ference, it was shown that every 10 mg/dl (21 nmol/l) Lp(a) increase above median is associated with a 5.8% relative risk increase for coronary artery disease (CAD) [4•]. Elevated Lp(a) is also associated with a high risk of ischemic stroke and heart failure as well as with cardiovascular and all-cause mortality, albeit with a smaller effect size. For patients with an Lp(a) above the 95th percentile compared to low levels, the HRs for ischemic stroke, cardiovascular mortality, and all-cause mortality were 1.6 [95%CI 1.2–2.1], 1.5 [95%CI 1.3–1.8], and 1.2 [95%CI 1.1–1.3] respectively, compared to 2.6 [95%CI 1.6–4.1] for myocardial infarction in the Copenhagen population studies [7–9]. In the same population, the HR for calcific aortic valve disease in patients with Lp(a) levels above the 95th percentile is 2.9 [95%CI 1.8–4.9], comparable to the HR for myocardial infarction [10].

Although convincing evidence has not been provided to date, most likely due to the lack of adequate animal models, multiple mechanisms have been identified through which Lp(a) may increase the risk of ASCVD or aortic valve stenosis. First, the Lp(a) particle contains apoB and may therefore have similar atherogenic properties as other apoB particles such as LDL, albeit that the absolute concentration of Lp(a) particles is usually much lower in comparison with LDL particles, even in patients with very high Lp(a) plasma levels. Second, Lp(a) is an important carrier of oxidized phospholipids which are recognized as damage-associated molecular patterns and therefore can result in pro-inflammatory as well as pro-calcific effects (in aortic valve stenosis). Third, it has been postulated that the apolipoprotein(a) part selectively binds to endothelial extracellular matrix proteins and thereby can be retained in the arterial wall [11]. Fourth, due to the plasminogen-like structure of apolipoprotein(a), Lp(a) is suspected to interfere with fibrinolysis, although only extremely elevated Lp(a) levels seem to be potentially associated with venous thrombosis in large-scale observational and genetic studies [12–14].

### Lp(a) Metabolism

Although the past decades of research into Lp(a) metabolism have provided numerous insights into Lp(a) synthesis, some aspects of the Lp(a) pathway still remain unclear. Remarkably, the exact location where the apolipoprotein(a) molecule is added to the apoB particle and how this molecule or the Lp(a) particle is subsequently cleared remains unknown. The major determinant of plasma Lp(a) concentration is the apo(a) kringle size, with larger kringle sizes leading to less efficient hepatic secretion and hence lower plasma levels [15]. It has been suggested that clearance of Lp(a) particles is achieved via the LDL receptor in the liver [16]. In line, homo- or heterozygous familial hypercholesterolemia patients with a (total) loss of the LDL receptor do have elevated Lp(a) plasma levels, although this may be caused by selection bias [17]. Nevertheless, the fact that statins do not lower Lp(a) but PCSK9 inhibitors do lower Lp(a), while both upregulate the LDL receptor, suggests clearance largely independent of the LDL receptor pathway. In addition, other lipid lowering therapies with a working mechanism not involving the LDL receptor pathway, such as niacin and CETP inhibitors, also lower Lp(a) plasma levels. Recent studies have shown that the apolipoprotein(a) molecule likely plays an important role in Lp(a) catabolism since apolipoprotein(a) isoform size is positively associated with the fractional catabolic rate [16].

In addition to hepatic clearance, the kidneys appear to contribute to clearance of Lp(a) as well. Kinetic studies have shown that apo(a) particles are excreted by the kidneys at a steady state. Already early in the process of chronic kidney disease (CKD), however, apo(a) excretion hampers and Lp(a) plasma levels increase [18, 19]. Hemodialysis patients have been shown to display a decreased Lp(a) clearance rate and elevated plasma levels as compared to healthy individuals [20, 21]. A higher Lp(a) concentration in the renal arteries than in the renal veins suggests direct renal involvement in Lp(a) catabolism [22]. Future kinetic studies will have to further uncover where and via which mechanisms Lp(a) is cleared.

## Lp(a) Lowering: in Whom and How Much?

Importantly, a person’s absolute ASCVD risk is the most important factor determining the magnitude of the absolute ASCVD risk increase due to Lp(a) elevation, reflecting a constant relative risk increase multiplying the baseline ASCVD risk of the patient. Hence, both in primary as well as secondary prevention, patients with high Lp(a) levels and high ASCVD risk are likely to benefit from Lp(a) lowering therapies. In secondary prevention patients, where there is a 10-year recurrence risk exceeding 20% [23], high Lp(a) levels have a major impact on absolute ASCVD risk. Sixty-three percent of patients is reclassified into a higher risk category of the SMART (Secondary Manifestations of ARTerial disease) risk score when Lp(a) levels are taken into account [24]. In addition, also primary prevention patients with very high Lp(a) levels could benefit from Lp(a) lowering. In a post hoc analysis from the primary prevention JUPITER (Justification for the Use of Statins in Prevention: An Intervention Trial Evaluating Rosuvastatin) trial, it was shown that even in patients with very low LDL-C levels (54 mg/dl) but still an elevated ASCVD risk due to increased CRP levels at baseline, Lp(a) plasma levels were also associated with an important residual ASCVD risk [25].

Unlike normally distributed LDL-C, Lp(a) distribution is skewed to the right, which results in a relative overrepresentation of patients with very high Lp(a) levels when compared to LDL-C distribution. Whereas guideline-recommended LDL-C lowering can be considered even in patients with relatively low LDL-C levels, Lp(a) lowering is likely to be restricted to those patients with high levels (above ~ 50–70 mg/dl) due to the large absolute reduction which is most likely needed to achieve significant impact on ASCVD risk. This absolute Lp(a) reduction which is needed has been estimated by several recent studies. In 2018, one of the Ference Mendelian randomization analyses in almost 200,000 patients conservatively estimated that a 102 mg/dl (213 nmol/l) reduction in Lp(a) would be needed to equal the ASCVD benefit from 1 mmol/l (38.67 mg/dl) LDL-C lowering [4•]. More recently, a less conservative observational analysis from the Copenhagen General Population Study by Madsen et al. in 58,527 patients estimated that a 50 mg/dl (105 nmol/l) lowering already equals the approximately 20% relative risk reduction achieved with 1 mmol/l LDL-C lowering [26]. Although discrepant, both studies underline the large Lp(a) reductions and thus high baseline Lp(a) levels needed for a clinically meaningful effect on ASCVD risk, despite the expected 80–90% potency of Lp(a) lowering therapeutics. Given their high absolute risk, secondary prevention patients could already benefit when Lp(a) levels exceed the clinically used threshold of 50 mg/dl. The Lp(a)-HORIZON (Assessing the Impact of Lipoprotein (a) Lowering With TQJ230 on Major Cardiovascular Events in Patients With CVD; NCT04023552) outcomes trial will elucidate the role of Lp(a) lowering in secondary prevention. To achieve a relevant ASCVD risk reduction in the primary prevention population, it is likely that baseline Lp(a) levels should be higher (e.g., 150 mg/dl) to achieve a clinically meaningful reduction. The precise threshold will have to be determined once the outcomes trials have finished.

## Approved Lipid Lowering Therapies and Lp(a)

To date, there are no approved specific Lp(a) lowering therapies yet; however, several already approved lipid lowering therapies have an effect on Lp(a) plasma levels (Table 1).

**Table 1:** Lipid lowering and Lp(a)-directed therapies

Drug/class name	Drug target	Development stage	Lp(a) reduction	LDL-C reduction	References
*Approved lipid lowering therapies*					
Statins	HMGCR	Available	No change	20–50%	[28, 47, 48]
Ezetimibe	NPC1L1	Available	0–7% (on top of statins)	18–22% (on top of statins)	[29, 30, 49]
Lipoprotein apheresis	Plasma lipoprotein removal	Available	63%	64%	[32]
Bempedoic acid	ACLY	Available	No change	17–21%	[34, 50–52]
PCSK9i monoclonal antibodies	PCSK9	Available	23–27% (on top of statins + ezetimibe)	50–60% (on top of statins + ezetimibe)	[35, 36]
Inclisiran	PCSK9	Available	22%	50%	[42]
*Lp(a)-directed therapies*					
Pelacarsen	ASO with GalNAc3 conjunction	Phase 3	80%	10–20%	[43, 44••, 53]
Olpasiran	siRNA	Phase 2	Up to 90%	No change	[45•]
SLN360	siRNA	Phase 1	Up to 98%	Up to 25%	[46•]

*Lp(a)*, lipoprotein(a); *PCSK9i*, proprotein convertase subtilisin kexin type 9 inhibiting; *HMGCR*, 3-hydroxy-3-methylglutaryl coenzyme reductase; *NPC1L1*, Niemann-Pick-like protein 1C1; *ACLY*, ATP citrate lyase; *siRNA*, small interfering RNA; *ASO*, antisense oligonucleotide; *GalNAc3*, N-acetylgalactosamine; *LDL-C*, low-density lipoprotein-cholesterol

### Statins

As foundation of LDL-C lowering and the prevention of ASCVD, statins are the most used lipid lowering drugs worldwide. While moderate- to high-intensity statins lower plasma LDL-C for 50% by upregulating the LDL receptor, statins do not lower plasma Lp(a) levels. In fact, a recent meta-analysis of 6 RCTs involving 5256 patients showed that statins significantly increased plasma Lp(a) levels by 11.6% to 24.2% compared to placebo [27]. In contrast, more recently, a much larger meta-analysis including 24,448 participants with individual patient data from 39 placebo-controlled RCTs showed no significant effect of statin treatment on Lp(a) plasma levels [28]. Therefore, it does not seem likely that plasma Lp(a) is affected in a clinically meaningful manner by statin therapy. In addition, even if statins would mildly increase Lp(a) levels, the ASCVD benefits from the LDL-C lowering will always outweigh the potential small increases in Lp(a) plasma levels.

### Ezetimibe

Multiple large trials and meta-analyses have investigated the impact of ezetimibe on Lp(a) levels. Whereas ezetimibe additionally lowers apoB and LDL-C plasma levels up to 20%, ezetimibe seems to have no or a very small effect on plasma Lp(a) levels. Both as monotherapy as well as in addition to statin therapy, ezetimibe had no effect on plasma Lp(a) concentration in a meta-analysis of 10 placebo-controlled RCTs including 5188 participants [29]. In contrast, a meta-analysis investigating ezetimibe monotherapy in 2337 patients from 7 RCTs illustrated a very small Lp(a) reduction of 7% [30]. Lp(a) lowering effects of this magnitude, if valid, will not have a clinical impact on ASCVD [4•, 26, 30],

### Lipoprotein Apheresis

Lipoprotein apheresis is primarily used in patients with homozygous familial hypercholesterolemia removing apoB lipoproteins. Since Lp(a) is also an apoB lipoprotein, Lp(a) levels are also significantly reduced upon apheresis therapy. In fact, apheresis is the only FDA-approved Lp(a) lowering treatment currently available. Especially in patients with very high Lp(a) levels and a residual high ASCVD risk despite maximally tolerated lipid lowering therapy, lipoprotein apheresis significantly reduces Lp(a) and most likely also ASCVD risk, although not investigated in an RCT [31, 32]. Lipoprotein apheresis is mainly used in Germany and the USA for Lp(a) lowering and has shown to reduce Lp(a) by 63% post-apheresis compared to pre-apheresis values, albeit transiently [32].

### Bempedoic Acid

The recently approved bempedoic acid is the third oral drug after statins and ezetimibe added to the lipid lowering armamentarium and additionally lowers apoB and LDL-C by approximately 20%, depending on the combination of lipid lowering therapies prescribed [33]. The scarce data available suggests that bempedoic acid has no relevant effect on Lp(a) plasma levels [34]. Considering that bempedoic acid inhibits ATP citrate lyase, just upstream of statin target 3-hydroxy-3-methylglutaryl coenzyme A (HMG-CoA) reductase, the effect on Lp(a) levels is presumably comparable with that of statins. The cardiovascular outcomes trial CLEAR Outcomes (Evaluation of Major Cardiovascular Events in Patients With, or at High Risk for, Cardiovascular Disease Who Are Statin Intolerant Treated With Bempedoic Acid) will likely provide large-scale clinical data to clarify whether bempedoic acid influences Lp(a) levels (NCT02993406).

### PCSK9 Inhibition

With the introduction of inclisiran, multiple therapies targeting proprotein convertase subtilisin/kexin type 9 (PCSK9) are available for lipid lowering. Both PCSK9 inhibiting monoclonal antibodies evolocumab and alirocumab have shown significant reductions in plasma Lp(a) levels in their phase III outcome trials. Evolocumab showed an Lp(a) reduction of 27% after 2.2 years of median follow-up in 25,096 patients from the FOURIER (Further Cardiovascular Outcomes Research with PCSK9 Inhibition in Subjects with Elevated Risk) [35], whereas alirocumab treatment resulted in a median Lp(a) plasma reduction trial of 23% after 2.8 years of follow-up in 18,924 patients in ODYSSEY Outcomes (Evaluation of Cardiovascular Outcomes After an Acute Coronary Syndrome During Treatment With Alirocumab) [36]. Importantly, in these studies, the relative and absolute Lp(a) reduction was strongly dependent upon baseline Lp(a) levels. In FOURIER, the relative Lp(a) reduction in patients in the top quartile of baseline Lp(a) was merely 16% compared to a much higher 28% in the other quartiles. Nevertheless, since these patients have much higher Lp(a) levels, the benefit in terms of MACE reduction which can be attributed to absolute Lp(a) lowering is greatest in patients with high Lp(a) levels as was suggested by two post hoc analyses [37, 38]. In both trials, the absolute MACE reduction was higher in patients with high baseline Lp(a) levels. In FOURIER, absolute MACE reduction was 2.4% in patients with Lp(a) levels above 50 mg/dl (105 nmol/L) compared to 1.4% in patients with levels below 50 mg/dl. In ODDYSEY Outcomes, the absolute MACE reduction in patients with Lp(a) levels in the highest quartile was 3.7% compared to only 0.5% in patients with Lp(a) levels from the lowest quartile [39]. In these 25% of patients with the highest Lp(a) values, 39% of the MACE benefit was attributable to Lp(a) lowering. Thus, it seems that PCSK9 inhibiting monoclonal antibodies could be used to attenuate the Lp(a)-mediated ASCVD risk in patients with very high Lp(a) levels.

More recently, the small interfering RNA agent inclisiran targeting PCSK9 mRNA has shown similar reductions in plasma apoB and LDL-C concentration as compared with PCSK9 inhibiting monoclonal antibodies [40–42]. In conjunction with additional LDL-C reductions of 50%, inclisiran resulted in Lp(a) reductions of approximately 26% and 19% in the ORION-10 and ORION-11 respectively [42]. Given the shared mechanism of action and approximately equal reductions in lipid fractions it is very likely that LDL-C and Lp(a) lowering with inclisiran will have similar effects on ASCVD risk as the PCSK9 inhibiting monoclonals. Therefore, inclisiran could also be used to reduce Lp(a)-mediated ASCVD risk in patients with very high Lp(a) levels, especially in those with expected difficulties in drug adherence.

## Lp(a) Lowering Therapies in Development

### Pelacarsen

Pelacarsen (TQJ230) is an N-acetylgalactosamine (GalNAc_3_) conjugated antisense oligonucleotide targeting apolipoprotein(a) mRNA [43]. The GalNAc_3_ conjugation ensures specific uptake by hepatocytes through the asialoglycoprotein receptor [43]. Pelacarsen is administered through a monthly subcutaneous injection. The early-phase clinical trials have shown promising and impressive results. The phase I/IIa trial proved pelacarsen to be safe and well-tolerated in 64 participants while reducing Lp(a) plasma levels [43]. The subsequent dose-ranging RCT in 286 patients with established ASCVD again showed a mean 80% reduction in plasma Lp(a) levels [44••]. Significant differences in adverse effects were limited to a higher frequency of mostly mild injection-site reactions compared to placebo (27% with pelacarsen vs. 6% with placebo) as well as a higher frequency of urinary tract infections (13% with pelacarsen vs. 6% with placebo). Currently, the cardiovascular outcomes trial Lp(a)-HORIZON has fully enrolled 7680 patients with established ASCVD and is estimated to be completed in 2025. This first outcome trial with a specific Lp(a) lowering compound will confirm whether pelacarsen treatment accomplishes MACE reduction, thereby determining the position of Lp(a) lowering in future lipid lowering guidelines.

### Olpasiran

Similar to inclisiran targeting *PCSK9*, olpasiran is an siRNA agent specifically targeting *LPA* mRNA [45•]. This GalNAc_3_ conjugated siRNA is administered subcutaneously less frequently: once in every 3 or 6 months. In the phase I trial in 64 healthy adults, Lp(a) was persistently reduced up to 90% without major safety issues [45•]. The phase 2 trial investigating 4 doses of AMG890 in 290 patients with ASCVD has finished recruiting and is expected to be completed in 2023 (NCT04270760).

### SLN360

Another GalNAc_3_ conjugated siRNA in early-phase clinical trials is SLN360. The first results from the phase I single ascending dose trial involving 32 participants showed an up to 98% reduction of Lp(a) with the highest dose while SLN360 was well-tolerated [46•]. Further early phase studies will provide more data regarding efficacy and safety (NCT04606602).

## Conclusion and Future Perspective

High Lp(a) levels are robustly and log-linearly associated with ASCVD and aortic valve stenosis. Above the 95th percentile, Lp(a) instigates an up to threefold increased risk of ASCVD and aortic valve stenosis. Until now, Lp(a) lowering options are scarce and the only option to partially lower Lp(a) levels are PCSK9 inhibitors, which are in general only reimbursed in high-risk and high residual LDL-C secondary prevention patients. New clinically tested Lp(a) lowering agents, however, may shift this paradigm. If phase 3 trials with these therapies show an ASCVD benefit, a major part of Lp(a)-mediated residual ASCVD burden can be abolished. With up to 90% plasma level lowering, the CVOT trial with pelacarsen which is due in 2025 will demonstrate whether this can be converted into ASCVD event reduction. Olpasiran and SLN360 (in phase 2 and phase 1 studies respectively) hold promise to achieve a similar or greater Lp(a) reduction with a lower dosing interval. In the future, therapy could be prescribed to high-risk patients (e.g., patients with familial hypercholesterolemia, chronic kidney disease, diabetes, and patients in secondary prevention) with elevated Lp(a) levels above 50 mg/dL. In patients with extremely high Lp(a) levels above approximately 150 mg/dL and thus a markedly increased lifetime CVD risk, Lp(a) lowering should be considered in primary prevention patients without other risk factors. The precise cut-off values and projected patient groups will depend on the clinical effectiveness and safety as well as reimbursement of Lp(a) lowering therapies. With the current therapies in clinical development, Lp(a) could transcend from an ASCVD risk modifier towards the new pathway to target in the upcoming decade.
